# Multi-Source Information Fusion Based on Negation of Reconstructed Basic Probability Assignment with Padded Gaussian Distribution and Belief Entropy

**DOI:** 10.3390/e24081164

**Published:** 2022-08-21

**Authors:** Yujie Chen, Zexi Hua, Yongchuan Tang, Baoxin Li

**Affiliations:** 1 School of Information Science and Technology, Southwest Jiaotong University, Chengdu 610097, China; 2 School of Microelectronics, Northwestern Polytechnical University, Xi’an 710072, China; 3 Qianghua Times (Chengdu) Technology Co., Ltd., Chengdu 610095, China

**Keywords:** Gaussian distribution, reconstructed basic probability assignment, Dempster-Shafer evidence theory, multi-source information fusion, belief entropy

## Abstract

Multi-source information fusion is widely used because of its similarity to practical engineering situations. With the development of science and technology, the sources of information collected under engineering projects and scientific research are more diverse. To extract helpful information from multi-source information, in this paper, we propose a multi-source information fusion method based on the Dempster-Shafer (DS) evidence theory with the negation of reconstructed basic probability assignments (nrBPA). To determine the initial basic probability assignment (BPA), the Gaussian distribution BPA functions with padding terms are used. After that, nrBPAs are determined by two processes, reassigning the high blur degree BPA and transforming them into the form of negation. In addition, evidence of preliminary fusion is obtained using the entropy weight method based on the improved belief entropy of nrBPAs. The final fusion results are calculated from the preliminary fused evidence through the Dempster’s combination rule. In the experimental section, the UCI iris data set and the wine data set are used for validating the arithmetic processes of the proposed method. In the comparative analysis, the effectiveness of the BPA determination using a padded Gaussian function is verified by discussing the classification task with the iris data set. Subsequently, the comparison with other methods using the cross-validation method proves that the proposed method is robust. Notably, the classification accuracy of the iris data set using the proposed method can reach an accuracy of 97.04%, which is higher than many other methods.

## 1. Introduction

Multi-source information fusion refers to the processing and fusion of data collected from diverse knowledge sources or sensors. It is now used in many fields such as fault diagnosis [1], life-cycle prediction of engineering parts [2], recommendation systems [3], and medical diagnosis [4], etc. The fusion algorithm for multi-source information must seriously consider the evaluation of different attributes because the impacts of different attributes on the fusion results may be diverse. However, information involved in fusion is often imperfect, mainly in terms of uncertainty, imprecision, incompleteness, ambiguity, multiplicity, conflict, etc. [5]. How to use multi-source information more efficiently has become a challenge. The techniques commonly applied to address uncertain information modeling and fusion include Bayesian estimation [6], fuzzy theory [7], Kalman filter theory [8], artificial neural network theory [9], DS evidence theory [10], etc.

Among the above methods, DS evidence theory enables representing and managing uncertainty without a priori information and expressing “uncertain” and “imprecise” information. By modeling the problem, DS evidence theory is able to process the data more appropriately in the fusion process, which can improve the accuracy of fusion and make the decision results more informative. DS evidence theory is widely applied by researchers in the multi-source information fusion field for classification [11,12,13], decision-making [14], and so on.

DS evidence theory was first proposed by Dempster [15] in 1967 to address the multi-valued mapping dilemma using upper and lower probabilities, and Dempster’s combination rule was also proposed in it. The DS evidence theory was further extended and refined by Shafer [16], who introduced the concept of trust function to form a “mathematical theory of evidence”. Nonetheless, there are shortcomings in DS evidence theory, especially for Dempster’s rule of combination [17,18,19]. For example, the inability to resolve situations of severe or complete conflicts of evidence. Conflict of evidence means that the evidence involved in the calculation supports conflicting results. Many works focus on this issue.

One is to investigate the determination methods of BPAs [20]. Researchers who study this perspective believe that using different BPA determination methods can make the BPAs obtained from raw data conversion contain more valid information, and it will be easier to obtain the correct fusion results subsequently [21]. The BPA determination methods are divided into function-based BPA determination and intelligent algorithm-based BPA determination. Among the function-based BPA determination methods, the triangular fuzzy function-based BPA construction method is the most employed owing to its simple construction [22,23]. In addition, there are methods to generate BPA using trapezoidal fuzzy functions [24], Gaussian fuzzy functions [25], etc. The function-based determination generally has the advantage of being simpler and less time-consuming to compute, but the loss or bias of information is larger. For intelligent algorithms, researchers use methods such as gray correlation function BPA [26] and kernel density estimation [27] to establish BPA. Intelligent algorithm-based BPA determination is better, but the complexity is often much greater than the combination rule, where computational cost and rewards are not well balanced.

The next perspective of improvement is the modification of Dempster’s combination rules, especially for the method of conflict evidence fusion. Researchers who have studied this point of view believe that this result arises due to the shortcomings of Dempster’s combination rule itself, which leads to discarding when processing conflicting data [28,29,30]. Yager [31], for example, eliminated the normalization process of Dempster’s combination rule and proposed a new combination rule that used coercion to assign highly conflicting information to the public, which reduced the impact of evidence conflicts, although this combination rule no longer guaranteed the associative law and the commutative law; Jiang and Zhan [30] proposed mGCR (modified generalized evidence theory), which made the combination result contain more obvious geometric features and the physical meaning of the original GCR; Smarandache and Dezert [32] proposed a new DSmT theory based on DS evidence theory, where the representation of evidence was no longer represented by a single BPA but consisted of an independent source of evidence and a related source of evidence, both of which were involved in the computation of the combination of the evidence. The strategy of modifying the rules of Dempster’s combination rules has been shown to be effective in some works. However, modifying the rules means that the new rules may result in the method no longer satisfying the constraints of the DS evidence theory. It is possible that the properties of evidence will change, which may lead to uncontrollable results.

The third perspective is to modify the evidence sources before fusing them to make them more reasonable logically [33,34]. Scholars believe that the problem mentioned arises from the drawback of evidence sources rather than combination rules. Murphy [35] obtained a preliminary-fused BPA by averaging the BPAs of multiple sources with the same focal element separately to achieve the reduction in conflict degree; Song et al. [36] composed a support matrix (SDM) between BPAs by means of Euclidean distance to take into account the associations and conflicts between the evidence. This method improves the accuracy and anti-interference ability of the combined results but is computationally complex. Weng et al. [37] argued that the degree of blurring of BPAs has become larger as the number of focal elements included increased. Therefore, a method of reconstructing the BPA was proposed to reflect the relationship between different focal element BPAs. By reassigning the BPAs, the uncertainty was reduced; Yin [38] proposed the negation of BPAs so that the uncertain information contained in the BPAs came from both positive and negative aspects to improve the accuracy of fusion. Moreover, Wu et al. [39] adapted DS evidence theory to tunnel collapse risk analysis. Wu et al. employed a normal cloud model, probabilistic support vector machines (SVM) and a Bayesian network to assign BPAs from statistical data, sensors and expert assessments, respectively. Moreover, the above BPAs were fused and participated in the calculation of Dempster’s combination rule. This approach achieved a high accuracy rate in assessing risk from multiple dimensions. However, its achievement was based on sacrificing a large amount of data collection and processing time, model training time, and computing time.

In this work, the DS evidence theory is modified from two perspectives: the determination of the initial BPAs and the evidence preprocessing. The main motivation is as follows:

1. Since the initial BPAs have a significant influence on the fusion results, Gaussian functions estimated by the maximum likelihood method are used for determining the initial BPAs. To enhance the generalizability of the method, we assume that the multi-source information involved in the fusion obeys a complex nonlinear joint distribution, and they are distributed normally. This hypothesis has proven to be valid and widely accepted [40]. Therefore, it is conventional to use Gaussian functions to build the initial BPA determination model. Furthermore, original data will be padded with the mean of the data correspondingly before being estimated by the maximum likelihood method in order to improve generalizability and mitigate overfitting due to the over-dependence on the provided data. The padding strategy was first used in mathematical statistics to supplement missing information or to reduce dimensionality [41,42]. Lopez-Martin et al. proved that embedding the features of samples into the mapping space was beneficial for improving the accuracy of detection [43]. They embedded sample labels in self-supervised learning networks to accomplish network intrusion detection.
2. To improve the ability to discern the uncertainty of information, a variety of methods are applied to extract more valid information from the original sources. Referring to Weng et al.’s method [37], the BPA is firstly reconstructed by assigning the original BPAs, and the BPAs’ values with high degrees of uncertainty are partially assigned to the BPA of the subset focal elements. Additionally, referring to Yin’s research [38] on the negation of BPA, the reconstructed BPA of the subset focal elements is improved by the negation of BPA to enhance the representation of BPA uncertainty information. We denote the result of the calculation after the above process as nrBPA. Such processing can reduce the uncertainty of BPAs while ensuring the uncertainty of BPAs, which makes the final information involved in DS fusion richer and can improve the accuracy of decision-making.
3. To reduce the impact of conflicting information from each source on the DS evidence fusion and to make the fusion results more robust. First, improved belief entropy is employed to measure the information entropy of information from each source. Then the initial fusion BPAs are calculated by the entropy weighting method based on the improved belief entropy, which will be involved in the subsequent Dempster’s combination rule calculation to obtain the results.

The steps to complete the multi-source information fusion using the proposed method can be divided into four steps. First, the initial BPAs are obtained using the multi-source information data set; secondly, the initial BPAs are reconstructed into nrBPAs through a series of normalization and uncertain information retention methods; in the third step, the improved belief entropy of nrBPAs is served as the information entropy. The inverse normalization results of information entropies are used as weights of mass function to synthesize several known pieces of evidence into preliminary-fused BPAs; finally, Dempster’s combination rule is used for accomplishing data fusion.

The remainder of the article is organized as follows. In the second part, some preparatory knowledge is briefly introduced. In the third part, a multi-source information fusion method based on DS evidence theory with a strategy of nrBPA and padded Gaussian BPA function is proposed. The fourth part numerically demonstrates this fusion method based on the UCI data set. The fifth part discusses the effectiveness of improving the fusion results and compares the performance with other evidence-theoretic-based methods using cross-validation. The sixth part draws conclusions.

## 2. Preliminaries

### 2.1. Dempster-Shafer Evidence Theory

DS evidence theory is a Bayesian theory-based uncertainty inference approach that integrates the upper and lower bounds of confidence of evidence by modeling information of different attributes [44] and completes data fusion using Dempster’s combination rule [15]. This section will introduce the basics of DS evidence theory briefly.

**Definition** **1.** *Define a finite, non-empty, mutually incompatible set of elements* $\Theta =\theta_{1},\;\theta_{2},\theta_{3}\ldots \theta_{i}\ldots \theta_{n}$*.* Θ *is called a frame of discrimination (FOD), where n is the total number of elements contained in *Θ*, and* $\theta_{i}\left(1\leq i\leq n\right)$ *are the elements belonging to* Θ*. There are* $2^{\left|▪\right|}$ *cases for all combinations of all elements belonging to *▪*, as shown in Equation (1).*

(1) $$2^{\left|▪\right|}=\left\{\emptyset ,\theta_{1},\;\theta_{2},\theta_{3}...\left\{\theta_{1},\;\theta_{2}\right\},\left\{\theta_{1},\;\theta_{3}\right\}...\left\{\theta_{1},\;\theta_{2},\theta_{3}\right\}\ldots ,\Theta \right\}$$

When analyzing evidence, it is necessary to establish an initial assignment of confidence to the evidence, which expresses the degree of support of the evidence for the proposition itself. In DS theory, it is accustomed to consider the confidence of evidence as the mass of a physical object, so the mass function is used for expressing the confidence of evidence, which is also called basic probability assignment or body of evidence.

**Definition** **2.** *Let A be an arbitrary subset of FOD and m(A) be the BPA of A. Then, the mapping* $2^{\left|▪\right|}\to \left[0,1\right]$ *satisfies the following properties.*

(2) $$\left\{\begin{array}{c} \sum_{A\subseteq \Theta }m\left(A\right)=1\\ m\left(\emptyset \right)=0 \end{array}\right.$$

*If* $m\left(A\right)>0$*, then A is said to be a focal element of m.*

**Definition** **3.** *For each A belonging to FOD* Θ*, the sum of its subsets of BPA is called the belief function* bel(A)*, which is used to express the probability that the result may be a subset of A. Let B be a focal element belonging to FOD* Θ*, and* bel(A) *is calculated as Equation (3).*

(3) $$bel\left(A\right)=\sum_{B\subseteq A}m(B)$$

**Definition** **4.** *For each A belonging to FOD* Θ*, the sum of all focal elements belonging to FOD* Θ *whose intersection with A is not empty is called the Plausibility function of A* Pl(A)*.* Pl(A) *is employed for expressing the maximum belief of proposition A. Let B be a focal element belonging to FOD* Θ*,* Pl(A) *is denoted as Equation (4).*

(4) $$Pl\left(A\right)=\sum_{A⋂B\neq \emptyset }m(B)$$

**Definition** **5.** *Let* $m_{1}$ *and* $m_{2}$ *be BPAs belonging to the same discriminative frame and independent of each other,* $B_{1}$*,* $B_{2}$*,* $\ldots B_{n}$ *and* $C_{1}$*,* $C_{2}$*,* $\ldots C_{m}$ *be all focal elements contained in* $m_{1}$ *and* $m_{2}$*, respectively, n is the number of focal elements in* $m_{1}$*, m is the number of focal elements in* $m_{1}$*. Suppose A is a single focal element belonging to the same discriminative frame, then according to the DS evidence fusion rule, we have Equation (5). With this calculation, only the BPAs of single focal element are retained*

(5) $$m_{1}⨁m_{2}\left(A\right)=\frac{1}{K}\sum_{B\cap C=A}m_{1}\left(B_{i}\right)\cdot m_{2}\left(C_{j}\right),\;0\leq i\leq n,\;0\leq j\leq m$$

*where* $K=\sum_{B\cap C\neq \emptyset }m_{1}\left(B_{i}\right)\cdot m_{2}\left(C_{j}\right)$ *is called the coefficient measuring conflict of* $m_{1}$ *and* $m_{2}$*.*

### 2.2. Negation of BPA

The traditional DS evidence fusion rule is susceptible to conflicting evidence, giving rise to counter-intuitive conclusions. Instead of traditional BPA, Yin et al., in 2018 [38] employed the modified negation of BPA to participate in fusion operations. Specifically, Yin et al. addressed the effect of negation on BPA by employing four uncertainty measures, which were confusion measure (Conf) [45], dissonance measure (Diss) [45,46], non-specificity (NS) [47], ambiguity measure (AM) [48], and aggregated uncertainty (AU) [49]. The experimental results showed that the negation process causes all five uncertainty measures of BPA to rise. As the negation process continued to iterate, the AU kept an increasing trend, and the other four factors fluctuated to different degrees. Finally, all five values converged to higher values than the original BPA. Therefore, we choose the negation operation to further process the BPA to obtain a higher uncertainty.

**Definition** **6.** *Suppose* $m_{A_{i}}$ *is the BPA on the FOD* Θ*, let* ${\bar{m}}_{A_{i}}$ *be complement of* $m_{A_{i}}$*, there exists* ${\bar{m}}_{A_{i}}=1-m_{A_{i}}$*. The modified negation of BPA is defined as Equation (6).*

(6) $${\bar{m}}_{A_{i}}=\frac{1-m(A_{i})}{2^{N}-2}$$

*In which* $N=\left|\Theta \right|$*, is the number of identification frames* Θ *containing all focal elements, and * $2^{N}-2$ *is the sum of the inverse of all BPAs on the identification frame* Θ*.*

### 2.3. Belief Entropy

#### 2.3.1. Deng Entropy

Shannon entropy is a common method to measure the inaccuracy of information by probability assignment, but in DS evidence theory, the uncertainty of evidence cannot be well measured.

**Definition** **7.** *Deng entropy* [50]*, proposed by Deng based on Shannon entropy, is defined as Equation (7).*

(7) $$E_{d}\left(m\right)=-\sum_{A\subseteq X}m\left(A\right){log}_{2}\frac{m(A)}{2^{|A|}-1}$$

*where A is a focal element of FOD* Θ*,* ∣A∣ *is a modulo operation on A, which is also equal to the number of elements contained in A. Deng entropy is a variant of the classical Shannon entropy, which decomposes m(A) by* $2^{|A|}-1$ *and is a means of measuring BPA uncertainty. When A is a single element, Deng Entropy degenerates to Shannon entropy.*

Yan and Deng pointed out in their paper [51] that Deng entropy does not characterize well the variability of BPAs containing different element types when they contain the same number of elements and assignments. To address this problem, Yan and Deng proposed the improved belief entropy inspired by the improvement of Deng entropy. By introducing the belief function, uncertainty can be distinguished when the mass function contains events of the same scale but with different elements. Improved belief entropy considers the information about the scale of the evidence and the relative size of the focal element with respect to the evidence.

**Definition** **8.** *Improved belief entropy is defined as Equation (8)*

(8) $$E_{Md}\left(m\right)=-\sum_{A\subseteq \Theta }m\left(A\right){log}_{2}\frac{m\left(A\right)+bel\left(A\right)}{2\left(2^{\left|A\right|}-1\right)}e^{\frac{\left|A\right|-1}{\left|X\right|}}$$

*where* bel(A) *is the belief function of A,* ∣A∣ *is the number of events contained in focal element A as shown in Equation (3).* ∣X∣ *is the number of non-empty events contained in BPA X.*

#### 2.3.2. Entropy Weight Method

The entropy weight method determines the weight of an index based on the definition of entropy in information theory. It is more objective, avoiding the subjectivity and blindness of setting weights artificially.

**Definition** **9.** *Suppose there are n sources of information, and the information entropies are* $E_{1},E_{2},E_{3}\ldots E_{n}$*; for example, we employ improved belief entropy* $E_{Md}$ *as information entropies in our works. Then, the weight of source i is calculated as Equation (9).*

(9) $$W_{i}=\frac{\frac{1}{E_{i}}}{\sum_{i=1}^{n}\frac{1}{E_{i}}}$$

### 2.4. Hypothesis Testing Based on Gaussian Probability Density Function

A probability distribution function describes the distribution pattern of values taken by a random variable. Parameter estimation is the process of estimating unknown parameters in the overall distribution based on random samples drawn from the overall population. The method of maximum likelihood estimation is a type of parameter estimation first proposed by the German mathematician C. F. Gauss in 1821, but the method is usually credited to the British statistician R. A. Fisher, who reintroduced the idea in his 1922 paper [52] and first explored some properties of this method. When we have an event occurrence in one trial, it is considered that the value at this time should be the one that makes the maximum of all possible values of t. The method of great likelihood estimation is to choose such a value of a parameter as an estimate of this parameter so that the selected sample appears in the selected overall probability as the maximum [53].

A large number of processes in the natural and social sciences naturally follow Gaussian distributions. Even if they are not inherently Gaussian distributed, Gaussian distributions often provide the best approximation. Therefore, Gaussian distribution is chosen to fit the distribution of information in this paper.

**Definition** **10.** *The Gaussian probability density function is described as Equation (10)*

(10) $$f\left(X\right)=\frac{1}{\sigma \sqrt{2\pi }}e^{-\frac{{\left(X-\mu \right)}^{2}}{2\sigma^{2}}}$$

*where X is a random variable obeying Gaussian distribution, μ is the expectation of the random variable X, and σ is the variance of the random variable X.*

The great likelihood method is used for constructing a Gaussian probability density function model for the random variable X. The specific implementation is based on a number of sample observations belonging to the random variable X. The expectation and variance of the two parameters of the Gaussian probability density function model are obtained.

**Definition** **11.** *Suppose* $X_{1},X_{2}\ldots X_{n}$ *are a set of independent samples of random variables X from a Gaussian distribution,* $x_{1},x_{2},\ldots x_{n},\left(n\in N^{*}\right)$ *are sample observations, the unknown parameter mean μ and variance σ in X are calculated as the following steps:*

*Firstly, the unknown parameter mean μ and variance σ likelihood function L is shown in Equation (11).*

(11) $$L\left(\mu ,\sigma^{2}\right)=L\left(x_{1},x_{2},\ldots x_{n};\mu ,\sigma^{2}\right)=\prod_{i=1}^{n}\frac{1}{\sigma \sqrt{2\pi }}e^{-\frac{{\left(x_{i}-\mu \right)}^{2}}{2\sigma^{2}}},1\leq i\leq n$$

*Solve* L(μ)*,* L(σ) *separately and take the value of zero after logarithmic partial derivative as in Equation*

(12) $$\left\{\begin{array}{c} \frac{\partial }{\partial \mu }\ln L=0\\ \frac{\partial }{\partial \sigma }\ln L=0 \end{array}\right.$$

*Finally, let Equation (12) be equal to 0, and the obtained are the maximum likelihood estimates* $\hat{\mu }$ *and* $\hat{\sigma }$ *of μ and σ. Substituting the likelihood function L into Equation (12), respectively, the final* $\hat{\mu }$ *and* $\hat{\sigma }$ *can be obtained as Equation (13).*

(13) $$\begin{array}{c} \hat{\mu }=\frac{1}{n}\sum_{i=1}^{n}x_{i}\\ {\hat{\sigma }}^{2}=\left(n-1\right)\sum_{i=1}^{n}{\left(x_{i}-\hat{\mu }\right)}^{2} \end{array}$$

## 3. Proposed Method

We propose a multi-source information fusion method based on the DS evidence theory with padded Gaussian BPA function and nrBPA. The method remedies the traditional DS evidence theory defects, including the inaccuracy of the calculation when the evidence conflicts severely or completely, the inability to recognize the uncertainty degree of BPA and the poor robustness.

To begin with, because the determination from the original BPA is the basis for the DS evidence theory, the determination results are closely related to the fusion results. Scholars have attempted in many ways to generate BPA to make it more useful for subsequent calculations, such as the method of fuzzy triangular affiliation function, interval generation, kernel function, etc. In our work, Gaussian functions with padding terms with mean values are utilized as the BPA functions. Complex distributions in reality are often close to Gaussian distributions, and such methods of fitting realistic distributions by means of Gaussian functions have also proven to be effective [40]. The comparison of the efficiency of our method with other determination methods is shown in Section 5.1.1. Inspired by the mean interpolation method in statistics, which is widely accepted to fill in defective data [41,42], we believe that when the amount of raw data is small, or incomplete, or jitter has a significant impact on the robustness of the method, overfitting is likely to occur. To improve the robustness of our method, the Gaussian functions are padded with mean data under a certain ratio. It makes the confidence level obtained closer to the mean value, so that the interference caused by some outliers is reduced and the overfitting of our method is alleviated. The effectiveness of this strategy will also be discussed in Section 5.1.2 based on the iris classification task. According to the outcome, we set the padding ratio to 40% as the default padding ratio of the method because this allows the method to guarantee better performance on both small and larger data sets (corresponding in the experiments as the ratio of samples participating in the training of the method) while ensuring that the BPA assignment model is determined by the information of the real data as much as possible. The padding ratio can be adjusted for different sizes of data sets for information fusion tasks in order to achieve better performance.

On the other hand, we believe that the degree of uncertainty and ambiguity of the evidence should be taken into account. The uncertainty of the evidence refers to the focal elements contained in the evidence. The greater the variety of focal elements contained in the evidence, the greater the uncertainty of the evidence, and the more possibilities for fusion results. Consequently, the uncertainty makes it easier to obtain correct fusion results. Therefore, we aim to find a representation that adequately reflects the uncertainty of BPA. Yin et al., proved the modified negation of BPA [38]. Based on the above viewpoint, we define a BPA representation: negation of reconstructed BPA, which is later abbreviated as nrBPA. First, the initial BPA is reconstructed using the method [37] by combining the degree of uncertainty of each BPA within the initial BPA, which both enhances the deterministic discriminative information and retains the uncertainty of the original BPA information. The degree of uncertainty of a BPA is defined as the number of focal elements contained in the BPA. The higher the number of focal elements, the vaguer the BPA is, and the lower the number of focal elements, the clearer the BPA is. The method [38] is then cited to generate the negation of reconstructed BPA. By considering the degree of dispersion of focal elements, more information was collected from both the positive and negative sides of BPA, and BPA becomes more uncertain. Moreover, it is pleasant that when the BPA degenerates to probability, the DS evidence will degenerate to a Bayesian distribution, and the negation of the BPA will also degenerate to the negation of probability. The result obtained from the above two steps is employed as the nrBPA. In addition, the difficulty of having 0 values in BPA using Gaussian BPA functions is discovered. BPAs are likely to obtain the same number of focal elements as all elements in FOD. This can lead to difficulties in measuring the uncertainty before different BPAs. Therefore, before performing Dempster’s combination rule, the improved belief entropy proposed by Yan and Deng [51] is referred to measure the lateral importance between heterogeneous sources of information. The improved belief entropy considers not only different totals but also variations in entropy values between BPAs with the same total but different elements, which is suitable for evaluating the nrBPAs.

**Figure 1:**
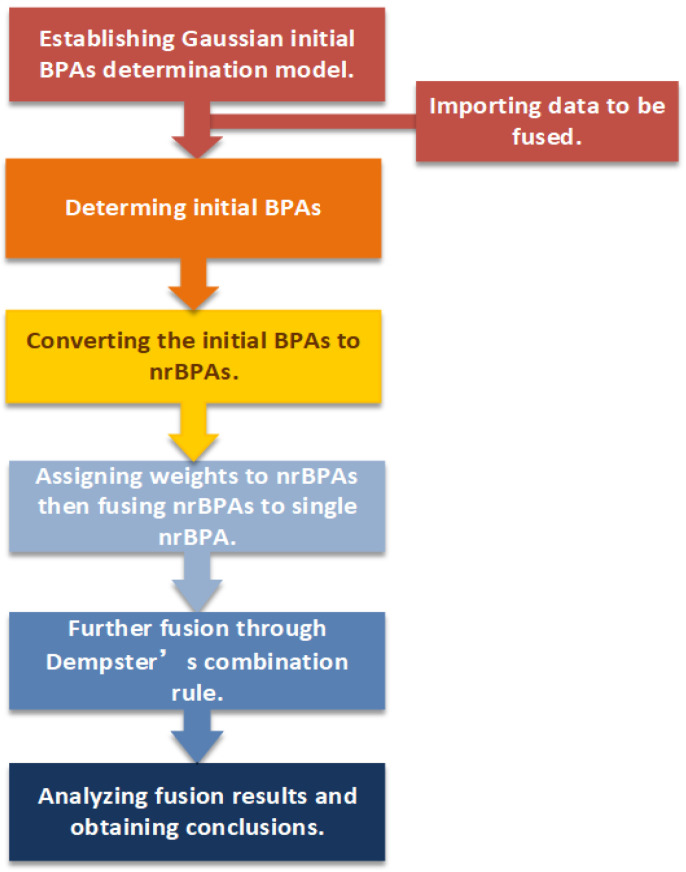
Flow chart for multi-source information fusion of the proposed method.

**Figure 2:**
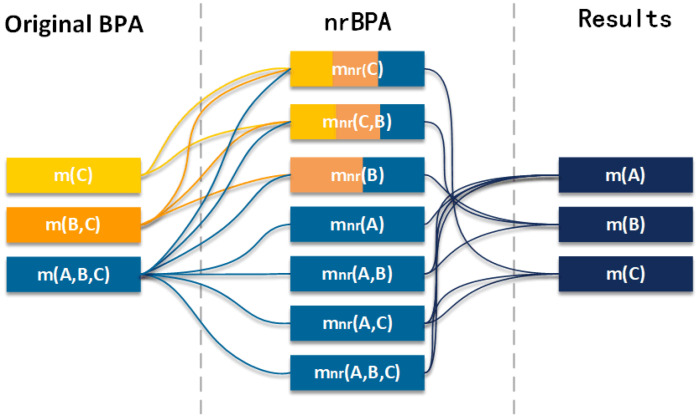
Schematic diagram of the reconstruction process of BPA.

In the proposed method, the first part is to construct a Gaussian BPA function. It is worth noting that besides the training data, each Gaussian function is padded with a certain percentage of data with the mean value of the training data to alleviate the over-fitting when the information in the data set is insufficient. The information to be fused is transformed into the initial BPAs by padded Gaussian BPA functions. After that, the initial BPAs are transformed into the nrBPAs, and the specific implementation process is divided into two steps. In the first step, the initial BPAs are reconstructed by assigning some values of the BPAs with high uncertainty to those with low uncertainty ones associated with them to reduce the uncertainty of the overall evidence. Since not all values of BPAs with high uncertainty are involved in the assignment, the type of focal elements contained in the evidence remains unchanged, and thus.,the uncertainty of the evidence is preserved; in the second step, the reconstructed BPAs are transformed in the way of negation. The negation of BPA caused the BPAs to contain increased uncertainty information from both positive and negative sides. Up to this point, nrBPAs have been generated. Again, the heterogenous nrBPAs are synthesized by the entropy weighting method into the preliminary fused BPAs. Finally, the final fusion results are obtained by Dempster’s combination rule using preliminary fused BPAs. The steps to achieve multi-source fusion using the method we proposed are shown in Figure 1. For ease of understanding, we show the change process of BPA in Figure 2. The detailed steps of the method are described as follows.

Step 1

Establishing the initial Gaussian BPA determination model. In order to transform the data into the initial BPAs, a Gaussian model was chosen, and the steps to build it are shown below.

**Step 1.1**. Obtaining the feature data set of known fusion results. The set of known fusion results $R=r_{1},{\;r}_{2}\ldots r_{O}$, which correspond to the identification framework θ in DS evidence theory, and $r_{1},{\;r}_{2}\ldots r_{O}$ are the fusion results, which correspond to the elements in DS evidence theory. The data set is represented as:

$$S\;=\left\{I_{1},\;I_{2}\ldots I_{N}\right\}$$

**Step 1.2**. Let *N* be the total number of data, the original data structure of each sample to be fused is assumed as:

$$I_{i}=\left\{s_{1},\;s_{2}\ldots s_{j}\ldots \;s_{M},{\;d}_{i}\right\},1\leq i\leq N,\;1\leq j\leq M.$$

where $s_{j}$ is each feature value, the last bit $d_{i}$ is the fusion result, ${\;d}_{i}\in R$, and *M* is the number of feature dimensions.

**Step 1.3**. The individual features of the training data are involved in estimating parameters $\hat{\sigma }and\hat{\mu }$ of the Gaussian function by the maximum likelihood method. Notably, in order to avoid overfitting of the generated Gaussian model, each feature is supplemented with a certain proportion of data with the value of the mean when calculating the variance. For example, if the original training data volume is N∗t, where *N* is the total, 0<t≤1 is the training proportion. For a feature, suppose the mean value of a certain event is μ, and the filling proportion is *p*, where 0≤p≤1. Then, (N∗t)∗p samples with the value of μ will be filled, and the size of the padded data set is (N∗t)∗(1+p).

Using the padded data set, the combination of the mean and variance of each feature on each category $\hat{\mu_{k}},\hat{\sigma_{k}}$ is calculated with reference to Equation (13). It is easy to obtain combinations of size M×O, constructed as $G=F_{1},F_{2},F_{3}\ldots F_{j}\ldots F_{M}$. *G* is the set of Gaussian probability density functions. $F_{j}=\left\{f_{1},\;f_{2}\ldots f_{k}\ldots f_{O}\right\}$, 1≤k≤O, is the set of Gaussian distributed probability density functions for each fusion result under the specified features.

Each $f_{k}$ is shown in Equation (14), which is obtained by substituting the corresponding combination of mean and variance into the Gaussian probability distribution function.

(14) $$f_{k}\left(X\right)=\frac{1}{\hat{\sigma_{k}}\sqrt{2\pi }}e^{-\frac{{\left(X-\hat{\mu_{k}}\right)}^{2}}{2{\hat{\sigma_{k}}}^{2}}}$$

Step 2

Determining the initial BPAs. The given data for each of the objects to be fused are input according to the structure $I=\left\{s_{1},\;s_{2}\ldots s_{j}\ldots s_{M}\right\}$. The obtained input data $I\prime =\left\{s_{1}\prime ,\;s_{2}\prime \ldots s_{j}\prime \ldots s_{M}\prime \right\}$ are substituted into the corresponding functions in the set of Gaussian probability density functions composed of Step 1.1, Step 1.2, and the initial BPAs can be obtained. Let the elements $r_{1},{\;r}_{2}\ldots r_{O}$ be sorted from smallest to largest by the values obtained after bringing in the corresponding probability density functions. $h_{1},h_{2},h_{3}$ are the values of the Gaussian functions of the feature values substituted into each fusion result, respectively, the corresponding BPAs are calculated as below.

$$m\left(r_{1},{\;r}_{2}\ldots r_{O}\right)=h_{1},$$

$$m\left(r_{2},\ldots r_{O}\right)=h_{2}-h_{1},$$

…

$$m\left(r_{O-1},r_{O}\right)=h_{O-1}-h_{O-2},$$

$$m\left(r_{O}\right)=h_{O}-h_{O-1}.$$

**Figure 3:**
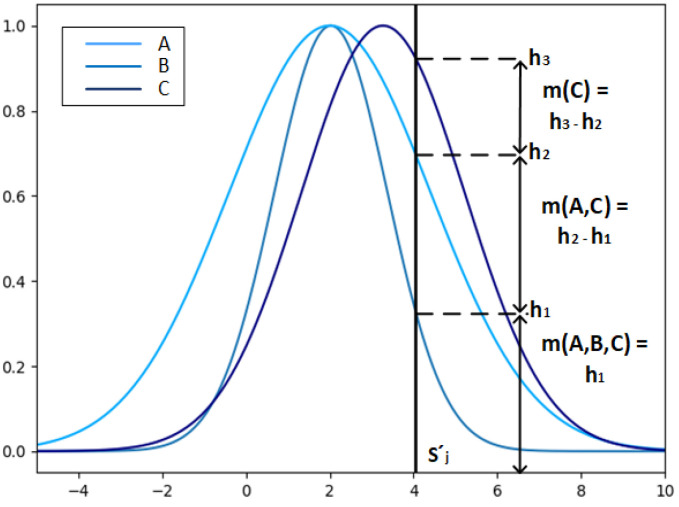
Schematic representation of BPAs determination by Gaussian BPA functions.

Let $r_{1}=B,{\;r}_{2}=A,r_{3}=C$, and the schematic diagram of the BPA calculation is shown in Figure 3. The horizontal coordinate value of the thick black line represents the feature value $s_{j}\prime$, and the intersection points $h_{1},h_{2},h_{3}$ are the intersection points of the feature value $s_{j}\prime$ and the Gaussian function of the fusion results B, A and C under the feature $s_{j}$, respectively, which determines the BPA about the feature value: values of *m*(*C*), *m*(*A*,*C*), *m*(*A*,*B*,*C*).

Step 3

Converting the initial BPAs to nrBPAs. The transformation of the original BPA to nrBPA is achieved using approaches from reference [37] and the method of reference [38]. The specific implementation steps are as follows.

**Step 3.1**. For a BPA, the more elements pointed to, the greater the uncertainty of that BPA and the more ambiguous the information contained. Weng et al.’s method [37] is proved to measure the uncertainty of BPA and reduce the information uncertainty. For all BPAs according to Equation (15).

(15) $$\left\{\begin{array}{c} m_{r}\left(A_{i}\right)=\sum_{A_{i}\subseteq A_{j}}\frac{m(A_{j})}{2^{|A_{j}|}-1}\;\;\;\;\;\;\forall A_{i},A_{j}\subset \Theta ,m\left(A_{i}\right)\neq 0\\ m_{r}\left(\Theta \right)=\frac{m(\Theta )}{2^{n}-1} \end{array}\right.$$

where $A_{i},A_{j}$ are the focal elements of $FOD\;\Theta ,\;|A_{j}|$ is a modulo operation on $A_{j}$, which is also equal to the number of elements contained in $A_{j},\;2^{|A_{j}|}-1$ represents the number of possible outcomes in $A_{j}$, which is a measure of uncertainty, and n is the number of focal elements contained in BPAΘ. With this operation, not only does each BPA’s data come from itself but from its upper sets, measuring the degree of association between individual BPAs. When the focal element of a BPA is BPAΘ, its only source of data is itself.

**Step 3.2**. The reconstructed BPAs are normalized according to Equation (16) in order to comply with the construction criterion of the BPA and to facilitate the subsequent operations.

(16) $$m_{r}\left(A_{i}\right)=\frac{m_{r}\left(A_{i}\right)}{\sum_{A_{j}\subseteq \Theta }m_{r}\left(A_{j}\right)}$$

**Step 3.3**. The reconstructed BPAs are transformed into nrBPAs, $m_{nr}$. By exploring both positive and negative information of the evidence through Yin et al.’s method [38], the inverse of the BPAs is obtained through Equation (6).

Step 4

The fusion results of heterogenous information are weighted using the entropy weighting method. The entropy weighting method has the ability to take the importance of heterogeneous sources of information into account. The specific steps are as follows.

**Step 4.1**. The uncertainties of BPAs are measured by improved belief entropy [51]. Equation (8) is applied to obtain the information entropy of each BPA, denoted as $E_{1},E_{2},E_{3}\ldots E_{M}$.

**Step 4.2**. Equation (9) is referenced to convert the information entropy into weights to obtain $w_{1},w_{2},w_{3}\ldots w_{M}$.

**Step 4.3**. The final BPAs of each focal element are obtained by multiplying the obtained BPAs with their corresponding weight value obtained by the entropy weight method and then multiplying the BPAs of different BPAs but the same focal element to obtain the final BPA of each focal element. Take the focal element $A_{i}$ belonging to BPAΘ as an example, *M* is the total number of features, and the final BPA A $m^{\prime }\left(A_{i}\right)$ is calculated as Equation (17).

(17) $$m^{\prime }\left(A_{i}\right)={w_{1}\cdot m}_{1}\left(A_{i}\right)+{w_{2}\cdot m}_{2}\left(A_{i}\right)\ldots w_{M}\cdot m_{M}\left(A_{i}\right)$$

Step 5

Further fusion through Dempster’s combination rule. The final BPA is combined M-1 times using the DS evidence theory combination algorithm, *M* is the total number of feature types, ⨁ denotes the calculation of Equation (5), and the fusion equation is as Equation (18).

(18) $$m\left(A_{i}\right)=m^{\prime }\left(A_{i}\right){\;⨁\;m}^{\prime }\left(A_{i}\right)\;⨁{\;m}^{\prime }\left(A_{i}\right)\;\ldots ⨁{\;m}^{\prime }\left(A_{i}\right)$$

Step 6

The fusion conclusion is obtained by comparing the combined results. Considering that the BPA was flipped by using negation, the smallest value is chosen as the highest confidence fusion conclusion.

## 4. Experiments

In this section, a series of experiments were elaborated on realistic data sets based on the methodology introduced. The performances of the method on given data sets are shown as well.

### 4.1. Demonstration of the Proposed Method

In this part, the classification tasks based on the UCI Iris data set [54] weree presented to show the process of the proposed method in the context of multi-source information fusion.

The iris data set contains 150 samples, 50 each from three species of iris-iris-setosa, iris-versicolor and iris-virginica. Each category contains four features-sepal length (SL), sepal width (SW), petal length (PL), and petal width (PW), where the first category of iris and the latter two categories of iris are linearly separable, while the latter two categories are linearly inseparable. For the convenience of representation, iris-setosa, iris-versicolor, and iris-virginica are abbreviated in the following formulas as A, B, C.

The proportion of data drawn from the data set employed for building the Gaussian distributed BPA generating function was referred to as the training proportion. As a preparation, we first disordered all the data and later randomly selected the data with 80% of the training proportion instead of using the proportional data within each data set, as this was more realistic. After that, these data were used for generating Gaussian distribution BPA to determine functions according to the great likelihood estimation. The padding proportion to 40% of the data with the values as the mean of the extracted data was set to alleviate overfitting. The mean and variance values of the Gaussian distributions obtained for the four features under the three iris types are shown in Table 1 below. In addition, all calculations were performed by a computer, and the results were accurate to seven decimal places. For convenience, all data are taken to three decimal places. This may lead to a slight difference in the results obtained during the operation between the displayed data and the data involved in the operation.

First, a random iris sample in the data set was selected with SL, SW, PL, and PW features and the ground truth as in Table 2.

**Figure 4:**
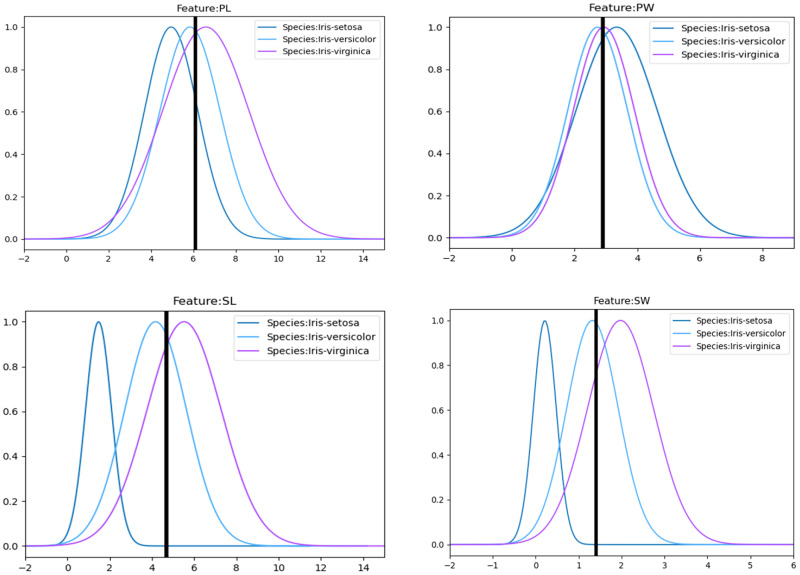
Eigenvalues and Gaussian distribution functions of the three irises under the corresponding SL, PL, SW, PW features. BPAs were generated based on the intersection of the eigenvalues with the Gaussian functions under the corresponding features.

The eigenvalues were substituted into the corresponding Gaussian distribution BPA determination functions to obtain the corresponding initial BPA, as shown in Figure 4. The Gaussian distribution BPA generating functions of the three iris types are drawn by curves of different colors, and the eigenvalues are marked by thick black lines, and the focal points of the thick black lines and the generating functions are the basis for the initial BPA determination.

The initial BPAs of each feature could be obtained according to Table 1. Then, the initial BPAs obtained under different features were shown below. It can be found that the generated BPA values were biased towards BPAs containing more focal elements with a higher degree of fuzziness, for example, (B, C, A) under SW feature reached 0.925.

$$S_{SL}:m\left(B\right)=0.039,\;m\left(B,C\right)=0.184,\;m\left(B,C,A\right)=0.777.$$

$$S_{SW}:m\left(B\right)=0.023,\;m\left(B,C\right)=0.051,\;m\left(B,C,A\right)=0.925.$$

$$S_{PL}:m\left(C\right)=0.120,\;m\left(B,C\right)=0.859,\;m\left(B,C,A\right)=0.0$$

$$S_{PW}:m\left(C\right)=0.177,\;m\left(B,C\right)=0.801,\;m\left(B,C,A\right)=0.001.$$

Afterward, in order to obtain nrBPAs, the BPAs were first reconstructed by Equation (15) to reduce the fuzziness of the BPA and obtain $m_{r}$. As an example, the calculation process of each BPA reconstruction for feature SL is shown below.

$$\begin{array}{cc} \; & m_{r}\left(A\right)=\frac{0.777}{2^{3}-1}=0.111.\\ \; & m_{r}\left(B\right)=0.039+\frac{0.184}{2^{2}-1}+\frac{0.777}{2^{3}-1}=0.212.\\ \; & m_{r}\left(\;C\right)=\frac{0.184}{2^{2}-1}+\frac{0.777}{2^{3}-1}=0.172.\\ \; & m_{r}\left(\;A,B\right)=\frac{0.777}{2^{3}-1}=0.111.\\ \; & m_{r}\left(\;A,C\right)=\frac{0.777}{2^{3}-1}=0.111.\\ \; & m_{r}\left(\;B,C\right)=\frac{0.184}{2^{2}-1}+\frac{0.777}{2^{3}-1}=0.172.\\ \; & m_{r}\left(\;A,B,C\right)=\frac{0.777}{2^{3}-1}=0.111. \end{array}$$

All the reconstructed BPAs are shown in Table 3. It can be seen that the BPAs with the highest uncertainty, such as *m*(*B*,*C*,*A*), were reduced, and the BPAs with low uncertainty, such as *m*(*B*), *m*(*C*), were increased.

Then, Equation (7) was applied to calculate the inverse of the reconstructed BPA, which results as nrBPA $m_{nr}$. The actual logic of the calculation was that when the number of focal elements contained in the BPA was 1, $m_{nr}$ was simply transformed into a difference relative to 1. When the number of focal elements was greater than 1, i.e., the degree of uncertainty was higher, the value obtained by dividing the number greater than 1 was smaller, and a smaller value can be obtained in the fusion result, which corresponds to the reinforcement of uncertainty of the evidence. The procedure of taking the negation of $m_{r}$ to obtain nrBPAs of feature SL are shown below.

$$\begin{array}{cc} \; & m_{nr}\left(A\right)=\frac{1-m(A)}{2^{1}-1}=0.148,m_{nr}\left(B\right)=\frac{1-m(B)}{2^{1}-1}=0.131,\\ \; & m_{nr}\left(C\right)=\frac{1-m(C)}{2^{1}-1}=0.138,m_{nr}\left(A,B\right)=\frac{1-m(A,B)}{2^{2}-1}=0.148,\\ \; & m_{nr}\left(A,C\right)=\frac{1-m(A,C)}{2^{2}-1}=0.148,m_{nr}\left(B,C\right)=\frac{1-m(B,C)}{2^{2}-1}=\;0.138,\\ \; & m_{nr}\left(A,B,C\right)=\frac{1-m(A,B,C)}{2^{3}-1}=0.148. \end{array}$$

The negation obtained from all reconstructed BPAs are shown in Table 4.

After obtaining the negation of BPA, the uncertainties of nrBPAs were measured by improved belief entropy through Equation (8). Later, the weight of each feature was calculated, according to the calculated information entropy by Equation (9). Taking the feature SL $E_{Md}\left(SL\right)$ as an example, the calculation is shown as follows:

$$\begin{array}{cc} \; & E_{Md}\left(SL\right)=-[0.148*\log\left(\frac{0.148+0.148}{2}e^{\frac{0}{3}}\right)+0.131*\log\left(\frac{0.131+0.131}{2}e^{\frac{0}{3}}\right)\\ \; & +0.138*\log\left(\frac{0.138+0.138}{2}e^{\frac{0}{3}}\right)+0.148*\log\left(\frac{0.148+0.148+0.148+0.131}{2*3}e^{\frac{1}{3}}\right)\\ \; & +0.148*\log\left(\frac{0.148+0.148+0.148+0.138}{2*3}e^{\frac{1}{3}}\right)\\ \; & +0.138*\log\left(\frac{0.138+0.138+0.131+0.138}{2*3}e^{\frac{1}{3}}\right)\\ \; & +0.148*\log\left(\frac{0.148+0.148+0.148+0.131+0.138+0.148+0.148+0.138}{2*7}e^{\frac{2}{3}}\right)]=1.852. \end{array}$$

When the number of focal elements increased, the improved belief entropy took the subset BPA data of BPA into consideration as well. The information entropy of all features could be obtained in the same way. The results are shown in Table 5.

According to Equation (9) weight of feature SL $w_{SL}$ was:

$$w_{SL}=\frac{\frac{1}{1.852}}{\frac{1}{1.852}+\frac{1}{1.846}+\frac{1}{1.868}+\frac{1}{1.870}}=0.251.$$

Similarly, the weights of all features are shown in Table 6.

Further, the BPAs of the four features were weighted and summed using the entropy weighting method Equation (18) to obtain the BPA $m_{w}$, and the BPAs of the species A $m_{w}\left(A\right)$ were calculated as follows. It can be found that the importance of different features for the fusion was distinguished by the entropy weights of the different features. Furthermore, in this example, feature SL, SW obtained higher weights. All the calculation results are shown in Table 7.

$$m\left(A\right)=0.148*0.251+0.145*0.252+0.167*0.249+0.167*0.249=0.157.$$

Finally, the BPAs of each category of the preliminary fusion were obtained using the DS evidence theory combination rule fusion $m_{w}$ The results are shown in Table 8, and the C with the smallest BPA value, i.e., iris-virginica, was selected as the classification result, which was the same as the ground truth.

### 4.2. Application to Realistic Classification Tasks

In this part, the proposed method is applied in real-world classification fusion tasks. Firstly, the proposed method uses the classification task of the UCI wine data set to validate the proposed method. The UCI wine data set [55] collects three types of wines with 13 attributes, namely alcohol, malic acid, ash, alcalinity of ash, magnesium, total phenols, flavanoids, nonflavanoid phenols, proanthocyanins, color intensity, hue, OD280/OD315 of diluted wines, and proline, with the number of samples in each category as shown in Table 9.

**Figure 5:**
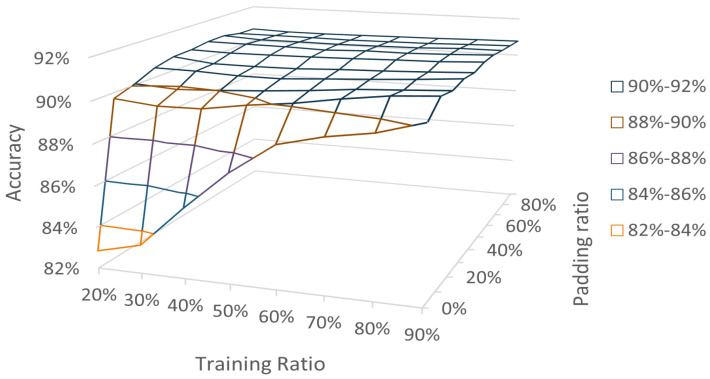
Accuracy under different training ratios and padding ratios in the wine classification task.

As per the results, our method achieved the highest average accuracy of 91.00% when the training ratio was 90% and padding was 60%. When the training ratio decreases, padding can also make the classification accuracy stabilize at a high level. When the amount of data is insufficient, using padding can classify more effectively as well. The relationship between the training set ratio, padding ratio and classification accuracy is shown in Figure 5, where each accuracy was obtained as the average value taken after 10 replicate experiments.

**Figure 6:**
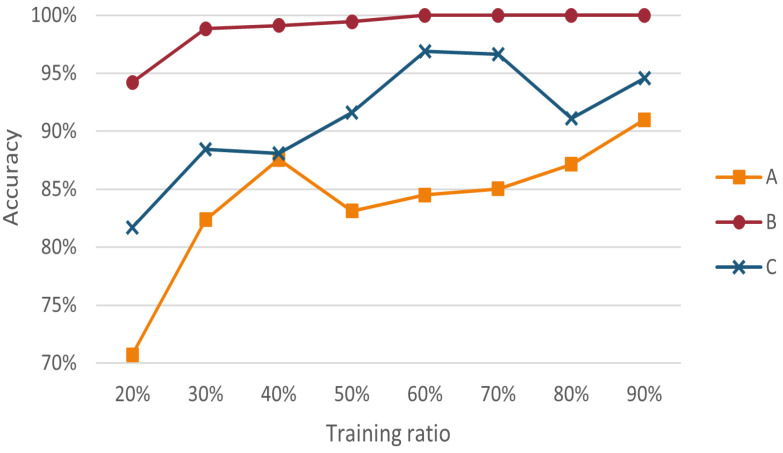
Accuracy of three types of wine with different training ratios.

Furthermore, the highest classification accuracy of the proposed method for different types was measured. The classification results for each type with a padding ratio of 20% and different training ratios were counted separately, as shown in Figure 6. In summary, when the training ratio was higher than 50%, the proposed method achieved a stable accuracy of 90–99% for both B and C and also achieved about 80% classification accuracy for A. When the training ratio was 60%, the classification accuracy values of B and C had reached over 95%, while the accuracy rate of A was in the rising stage. When the training ratio reached 90, the classification accuracy for class A improved to over 90%.

Furthermore, we also applied the proposed method to the breast cancer data set [56] and dry beans data set [57] classification tasks. Including the previously introduced data sets, the iris data set [54] and wine data set [55], the results are shown in Table 10. The validation method used is k-fold cross-validation, which will be described in detail in Section 5.2.

## 5. Comparative Analysis

In this chapter, the validity of the improvements and the robustness of the method were validated by a series of means. The iris data set from UCI was used for completing this part of the validation. It should be noted that Section 4.1 has a slight difference in the values obtained since the data extraction method used is a random sampling of a certain percentage of data from within all species; therefore, the fusion results of the method may differ in the effects of BPA determination due to the different order of arrangement of the data read in each experiment.

### 5.1. Discussion on Effectiveness of the Improved Method

The effectiveness of using the Gaussian function to determine the BPA and padding the mean terms when constructing the Gaussian distribution were discussed, respectively. The training data set for each classification task in this section was obtained by performing both data set disruption and random sampling. Furthermore, the accuracy is the average accuracy obtained by conducting each group of experiments ten times.

#### 5.1.1. Discussion on Effectiveness of Using Gaussian BPA Function

The discussion on the effectiveness of using a Gaussian probability distribution function to determine BPAs. We learned that some papers [22,23] used the triangular fuzzy function to accomplish this work, and the fusion performance of this method was compared. For determining the BPA using a triangular affiliation function, each feature contains a triangular affiliation function for each category, assuming that the category is A and the minimum, average and maximum values of the features under category A are $a_{1},a_{2},a_{3}$, respectively, the trigonometric function is denoted as $A=(a_{1},a_{2},a_{3}))$, and the BPA generation stage obtains the deployed by projecting the original feature values into the trigonometric function BPA.

**Figure 7:**
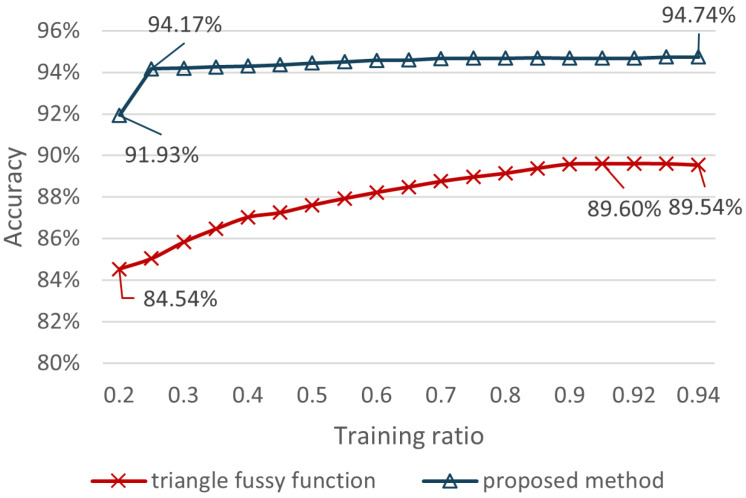
Accuracy of BPA determination based on the proposed method and triangle fussy function.

The comparison experiments between these two approaches were accomplished under the condition of ensuring the same means of subsequent fusion processing. In each experiment, data were randomly selected with ratios of 20%, 25%, 30%, 35%, 40%, 45%, 50%, 55%, 60%, 65%, 70%, 75%, 80%, 85%, 90%, 92%, and 94% from data set as the training set, respectively, and the remaining data were used for the test set. The accuracy graphs of the two methods are shown in Figure 7. Both methods show an increasing trend in accuracy as the training ratio rises. At a training ratio of 20%, the triangular fuzzy function and the proposed method possessed a base accuracy of 84.54% and 91.93%, respectively. In contrast to the triangular fuzzy function, which achieved a maximum accuracy of 89.60% at 90% of the training ratio, the accuracy of the proposed method stabilized at 94.17% to 94.74% when the training ratio was higher than 25%, and the maximum accuracy was 94.74%, which was 5.14% higher than that of the triangular fuzzy function. In conclusion, the proposed method is more stable and more accurate than the triangular fuzzy function.

#### 5.1.2. Discussion on Effect of the Padding Strategy for Generating BPA Function

**Figure 8:**
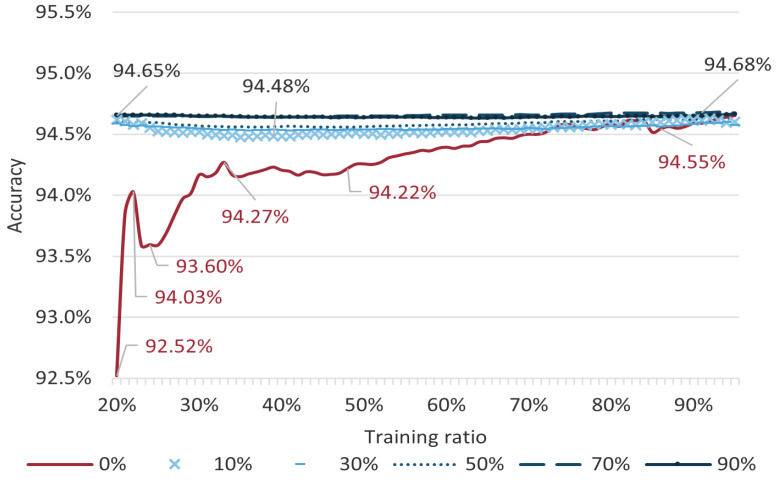
Accuracy of the proposed method with different padding ratios on different training ratios.

The discussion on the effect of using the padding strategy for generating the Gaussian BPA function. In the proposed method, the data used for generating the BPA determination function is composed of the training data and a certain percentage of the mean padding terms of the training data. We completed the discussion through the iris classification case. The accuracy of the proposed method obtained at training ratios from 20% to 100% with padding ratios of 0%, 10%, 30%, 50%, and 70% is shown in Figure 8. It can be seen intuitively that the method with padding terms had higher accuracy when the data volume was in the range of 20% to 70%, and the classification accuracy obtained by this method was more stable. Because the setting of the padding will make the functions used for Gaussian BPA determination tend to give higher confidence values for values that are in the vicinity of the mean of the corresponding features. The results prove that this method can improve the stability and accuracy of the multi-source information fusion system when the data are insufficient. It is not rare for training data to be inadequate in real-life multi-source information fusion tasks caused by small or under-informed data sets. With a capacity of 150, the iris data set classification task, in fact, also becomes a classification task based on a small data set.

### 5.2. Discussion on Robustness

Since the iris data set itself is a data set from reality, it is used it as a robustness examination. The main instrument employed in this section to compare the differences between the various methods is cross-validation. Cross-validation, proposed by Geisser S [58] and sometimes named rotation estimation, is a common validation method in statistics and machine learning. It achieves the effect of maximizing the data by selecting different parts of the data set each time and is suitable for scenarios where the size of the data set is small such that the training and test sets cannot be completely separated to complete model validation, which is similar to ours.

In particular, we use the k-fold cross-validation in cross-validation, where k = 10, as follows:

1. Dividing all data sets into 10 parts;
2. The model is completed by taking one of the test sets without duplication and using the other nine as training sets. After that, the accuracy $A_{i}$ of the used method on the test set is calculated. Positive samples with correct classification are set as true positive examples (TP), positive samples with incorrect classification are set as false positive examples (FP), negative samples with correct classification are set as false positive examples (FP), and the formula for the accuracy A is given in Equation (19).
  (19) $$A=\frac{TP+TN}{FP+TP+FN+TN}$$
3. Averaging the 10 accuracies to obtain the final accuracy rate, as shown in Equation (20).
  (20) $$A_{\left(10\right)}=\frac{1}{10}\sum_{i=1}^{10}A_{i}$$

Contrary to the previous experiments, the training data for each classification task in this chapter are obtained by taking the corresponding proportion of each feature from the randomly disrupted data set evenly.

**Figure 9:**
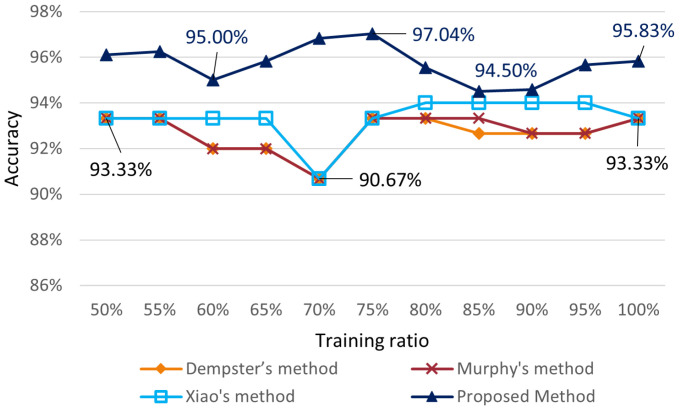
Accuracy of different methods with different training ratios on the iris data set.

We first used 150 samples from the iris data set as a training set to conduct k-fold cross-validations. The proposed method’s padding ratio for the Gaussian distribution BPA generating function was set at 40%. Algorithms involved in the comparison were Dempster’s method [15], Murphy’s method [35] and Xiao’s method [59]. The classification results obtained for training set ratios from 50% to 100% are shown in Figure 9, where the classification results of Dempster’s method and Murphy’s method and Xiao’s method were from the paper [59]. At a training ratio of 50%, the accuracy of Dempster’s, Murphy’s, and Xiao’s methods was 93.33%, while the proposed method could already reach 96.11% accuracy. When the training ratio reached 60%, the accuracy of the proposed method slightly decreased to 95%, Xiao’s method kept maintaining the accuracy at 93.33%, and both Dempster’s and Murphy’s methods dropped to 92.00%. During the training ratio from 60% to 70%, the accuracy of all three methods involved in the comparison dropped to 90.67%, while the accuracy of the launched method continued to rise to 96.82%, which indicated that the launched method had strong robustness. When the training ratio was 75%, the accuracy of the other three methods involved in the comparison rebounded to 93.33% at 75%, while the accuracy of the proposed method reached a maximum of 97.04% at that time. The accuracy of each method changed more gradually between 80% and 100% of the training ratio, with the accuracy of the proposed method stable between 95.57% and 94.50% and the accuracy of the other three methods stable between 94.00% and 92.67%. Overall, the accuracy of the proposed method was always above the other three methods involved in the comparison during the change in the training percentage from 50% to 100%, and the proposed method could maintain a flatter change trend when the other methods showed a sudden drop, which indicated that the proposed method had better robustness.

**Figure 10:**
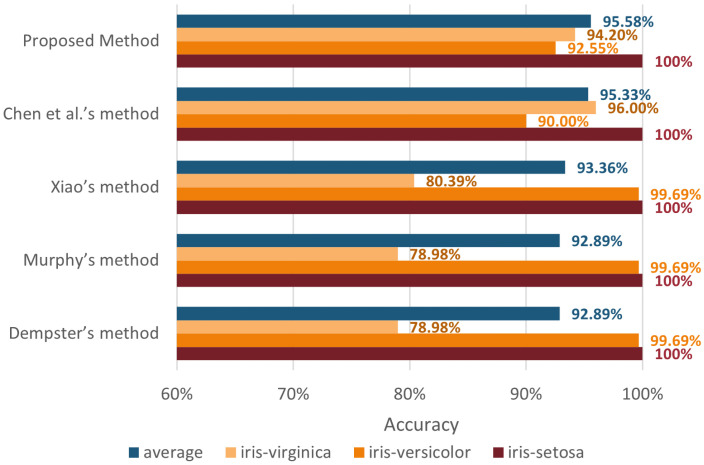
The accuracy of five ways to classify three species of iris.

The classification accuracy of the proposed method for each species of iris was compared with the results of Dempster’s method [15], Murphy’s method [35], Xiao’s method [59], and Chen et al.’s method [60]. The results are shown in Table 11 and Figure 10, respectively. It can be found that all five methods could achieve 100% accuracy in iris-setosa. Dempster’s, Murphy’s, and Xiao’s methods all have a higher accuracy of 99.69% in iris-versicolor classification, but only obtained an accuracy of 78.98% to 80.39% in the iris-virginica category. Chen’s method was able to achieve accuracy of 90% and higher accuracy in all species’ classifications than Dempster’s method, Murphy’s method and Xiao’s method. However, the average accuracy of Chen et al.’s method was lower compared to the proposed method. The variance of the accuracy of the proposed method was 0.001, which was the smallest among the five compared methods. The comparison indicates that the proposed method has better stability in multi-source information fusion.

The best performances of Dempster’s method [15], Murphy’s method [35], Xiao’s method [59], Chen’s method [60] and the proposed method were tested on the classification task of the iris data set. In addition to the above methods based on evidence theory, the KNN-based method [61] and deep neural network-based method [62] were also involved in the comparison, and the results are shown in Table 12. The proposed method was able to achieve a maximum accuracy of 97.04%, which is higher than the other four algorithms that participated in the comparison.

## 6. Conclusions

This paper proposes a new approach for multi-source information fusion in the frame of DS evidence theory. Gaussian functions with padding terms to determine BPAs were shown to be effective in alleviating the problem of over-fitting. It enables the use of the method when there is insufficient information. For measuring the uncertainty of BPA well, a new BPA representation—rnBPA—is proposed, which allows the clear BPA’s value to be enhanced while uncertainty evidence is ensured and collects the potential information contained in the BPA. In the experiments, we illustrated how the proposed method works with classification tasks based on the UCI iris data set, a wine data set, a breast cancer data set and dry beans data set. For comparative analysis, a comparison of the effect between the triangular fuzzy and the Gaussian function-based BPA and the discussion on the positive effects of padding terms in Gaussian BPA functions were designed to verify the superiority of BPA functions utilized in this work. It is experimentally demonstrated that the application of Gaussian distribution with padding terms enables the fusion method to be effective. After that, we used the cross-validation method to compare the effects of different data fusion methods on the classification task of the UCI iris data set. The launched method obtained a stable accuracy of above 94%, which shows superior robustness. With the highest accuracy of 97.04%, the proposed method won the best accuracy in comparison to many other methods. For limitations, we assumed that the data in this work is close to a normal distribution, which is useful for uniformly selected datasets and was proven to be effective in the experiment. However, if the dataset has high atypicality, it can lead to inaccurate results. As a result, further research on improving the method under high bias, such as optimizing the initial BPA building model, is worthwhile. In addition, we found that in the application to wine classification Section 4.2, type B accounted for nearly 40% of the dataset and maintained a high level of accuracy in the classification results, while the accuracies of the other two types were more volatile. That was possibly caused by the fact that the factor method did not take certain measures to give enough attention to the categories with low particle size, which also needs further discussion.

## Figures and Tables

**Table 1:** μs, σs of different kinds of features obtained through the training set.

Parameters	Category	SL	SW	PL	PW
μ	iris-setosa	4.983	3.393	1.478	0.243
	iris-versicolor	5.950	2.796	4.261	1.322
	iris-virginica	6.566	2.989	5.532	2.030
σ	iris-setosa	1.267	1.302	0.678	0.373
	iris-versicolor	1.782	1.093	1.717	0.744
	iris-virginica	2.345	1.286	2.010	1.094

**Table 2:** Features and ground truth of the selected sample.

SL	SW	PL	PW	Ground Truth
5.9	3.0	5.1	1.8	iris-virginica

**Table 3:** Reconstructed BPAs of the selected sample.

	*m*(*A*)	*m*(*B*)	*m*(*C*)	*m*(*A*,*B*)	*m*(*A*,*C*)	*m*(*B*,*C*)	*m*(*A*,*B*,*C*)
SL	0.111	0.212	0.172	0.111	0.111	0.172	0.111
SW	0.132	0.149	0.173	0.132	0.132	0.149	0.132
PL	0.000	0.293	0.415	0.000	0.000	0.293	0.000
PW	0.000	0.273	0.454	0.000	0.000	0.273	0.000

**Table 4:** NrBPAs of the selected sample.

	$m_{\mathrm{nr}}$(A)	$m_{\mathrm{nr}}$(B)	$m_{\mathrm{nr}}$(C)	$m_{\mathrm{nr}}$(A,B)	$m_{\mathrm{nr}}$(A,C)	$m_{\mathrm{nr}}$(B,C)	$m_{\mathrm{nr}}$(A,B,C)
SL	0.148	0.131	0.138	0.148	0.148	0.138	0.148
SW	0.145	0.142	0.138	0.145	0.145	0.142	0.145
PL	0.167	0.1184	0.098	0.167	0.167	0.118	0.167
PW	0.167	0.121	0.092	0.167	0.167	0.121	0.167

**Table 5:** The information entropy of all features of the selected sample.

$E_{\mathrm{SL}}$	$E_{\mathrm{SW}}$	$E_{\mathrm{PL}}$	$E_{\mathrm{PW}}$
1.852	1.846	1.868	1.870

**Table 6:** The weights of all features.

$W_{\mathrm{SL}}$	$W_{\mathrm{SW}}$	$W_{\mathrm{PL}}$	$W_{\mathrm{PW}}$
0.251	0.252	0.249	0.249

**Table 7:** Weighted BPAs $m_{w}$ of the selected sample.

$m_{w}$(A)	$m_{w}$(B)	$m_{w}$(C)	$m_{w}$(A,B)	$m_{w}$(A,C)	$m_{w}$(B,C)	$m_{w}$(A,B,C)
0.157	0.128	0.116	0.157	0.157	0.130	0.157

**Table 8:** Fusion results of the selected sample using the proposed method.

*m*(*A*)	*m*(*B*)	*m*(*C*)
0.628	0.230	0.141

**Table 9:** Number of samples in each category of wines.

A	B	C
59	71	48

**Table 10:** Accuracies of the proposed method with different data sets.

Data Set	Accuracy
Iris [54]	97.04%
Wine [55]	95.37%
Breast Cancer [56]	94.90%
Dry Beans [57]	86.89%

**Table 11:** Comparison of the classification accuracy on each category, mean accuracy and variance of the proposed method with other methods.

	Iris-Setosa	Iris-Versicolor	Iris-Virginica	Average	Variance
Dempster’s method [15]	**1.0000**	**0.9969**	0.7898	0.9289	0.0097
Murphy’s method [35]	**1.0000**	**0.9969**	0.7898	0.9289	0.0097
Xiao’s method [59]	**1.0000**	**0.9969**	0.8039	0.9336	0.0084
Chen et al.’s method [60]	**1.0000**	0.9000	**0.9600**	0.9533	0.0017
Proposed Method	**1.0000**	0.9255	0.9420	**0.9558**	**0.0010**

**Table 12:** Comparison between the best performances of the proposed method with other methods.

Method	Accuracy
Dempster’s method [15]	92.89%
Murphy’s method [35]	92.89%
Xiao’s method [59]	94.00%
Chen et al.’s method [60]	95.47%
KNN-based method [61]	96.67%
deep neural network-based method [62]	96.00%
Proposed method	**97.04%**

## Data Availability

All data generated or analyzed during this study are included in this published article.
